# Primary renal angiosarcoma with extensive hemorrhage: CT and MRI findings

**DOI:** 10.1590/S1677-5538.IBJU.2018.0375

**Published:** 2019-04-01

**Authors:** Suk Hee Heo, Sang Soo Shin, Taek Won Kang, Ga Eon Kim

**Affiliations:** 1 Department of Radiology, Chonnam National University Medical School, Gwangju, South Korea;; 2 Department of Urology, Chonnam National University Medical School, Gwangju, South Korea;; 3 Department of Pathology, Chonnam National University Medical School, Gwangju, South Korea

**Keywords:** Magnetic Resonance Imaging, Tomography, X-Ray Computed

## Abstract

Primary angiosarcomas of the kidney are very rare, but highly aggressive tumors showing poor prognosis. Patients frequently complain of flank pain, hematuria, or a palpable mass. We present a case of primary renal angiosarcoma occurring in a 61-year-old man. CT images depicted a huge exophytic mass (16 cm in diameter) in the right kidney, exhibiting extensive hemorrhage. The mass showed centripetal peripheral nodular enhancement on dynamic contrast-enhanced images. Furthermore, MR imaging revealed a tangled mesh of tumor vessels in the periphery of the mass. We suggest its inclusion in the differential diagnosis of cases of hemorrhagic renal tumors with prominent vasculature.

## INTRODUCTION

Primary renal angiosarcomas are exceedingly rare, but highly aggressive tumors showing poor prognosis (1). The etiology of primary angiosarcomas of the kidney has not yet been elucidated (2, 3). These tumors are predominantly found in older men (60-70 years of age) (2, 3). Due to the low incidence of this tumor, angiosarcoma is not usually considered in the diagnosis of renal tumors associated with retroperitoneal hemorrhage (4). In this report, we describe a case of primary renal angiosarcoma showing extensive hemorrhage with an emphasis on imaging features using dynamic CT and MRI.

## CASE REPORT

A 61-year-old man was admitted to our hospital with a hematoma in the right kidney. This diagnosis had been made 20 days prior to his admission. His clinical symptoms included pallor and anemia, but physical examination revealed no rigidity or distension of the abdomen; however, he complained of discomfort on palpation in the right flank area. The initial routine laboratory tests showed the hemoglobin level 10 g / dL (normal range: 12-18) and platelet count was 85 x 103 / mm^3^ (normal, 130-450). The serum levels of blood urea nitrogen and creatinine, and urinalysis were within normal limits.

**Figure 1:**
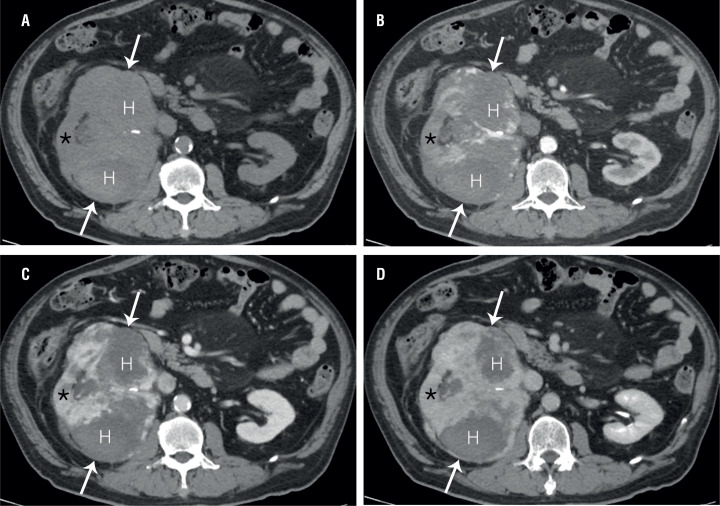
Axial CT images of pre-contrast (A), contrast-enhanced corticomedullary (B), nephrographic (C) and excretory (D) phases depict a huge mass (arrows) with extensive hemorrhage (H) in the right kidney, which shows progressive peripheral nodular enhancement with a delayed fill-in. Note the presence of residual renal parenchyma (*).

**Figure 2:**
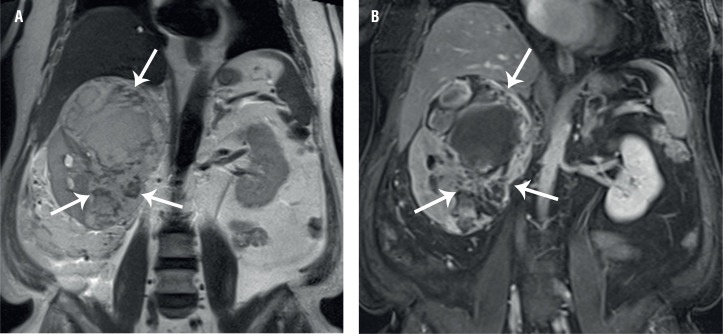
Coronal T2-weighted (A) and fat-suppressed contrast-enhanced T1-weighted (B) MR images demonstrating a tangled mesh of signal-void tumor vessels (arrows in A) in the periphery of the mass, corresponding to the areas with strong enhancement (arrows in B).

CT images (Figure-1) depicted a huge exophytic mass, measuring 16 cm in diameter, in the right kidney. The mass also exhibited extensive hemorrhage. The mass showed peripheral nodular enhancement, as shown on cortico-medullary-phase CT, accompanied by delayed centripetal filling on nephrographic and excretory-phase CT images. Meanwhile, MRI (Figure-2) demonstrated a tangled mesh of tumor vessels with signal voids in the periphery of the mass on coronal T2-weighted images, corresponding to the areas with strong enhancement on contrast-enhanced coronal MR images. There were no additional mass lesions observed in other solid organs in the abdomen. Based on these imaging findings, the differential diagnoses included hemangioma, angiosarcoma, and angiomyolipoma.

**Figure 3:**
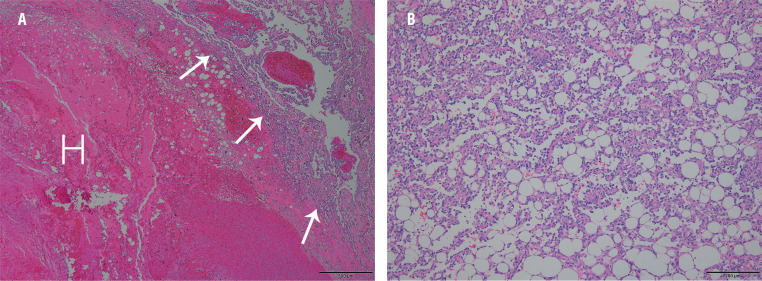
Histological sections reveal hematoma (H) and anastomosing channels with obvious vasoformation and dissecting growth pattern (arrows) (A: H & E stain, x 40) and complex anastomosing channels with endothelial papillae (B: H & E stain, x 100).

Using a transperitoneal approach, the patient underwent a radical right nephrectomy. The macroscopic appearance showed a huge mass in the right kidney that extended up to the perirenal space. The mass showed extensive hemorrhage and proliferation of the tumor vessels. The microscopic features revealed complex anastomosing channels with obvious vasoformation and endothelial papillae (Figure-3). Immunohistochemical stains tested positive for ERG, CD 34, CD 31, and Ki-67. To make a differential diagnosis, we considered the pathologies of various vascular tumors, including angiosarcomas, hemangiomas, and hemangioendotheliomas. A final diagnosis of a primary renal angiosarcoma was made based on the aforementioned histological features.

## DISCUSSION

Angiosarcoma is an aggressive malignant neoplasm originating from endothelial cells of the blood and lymphatic vessels (5, 6). Among the various malignant tumors that can occur in the kidney, primary angiosarcomas are extremely rare, with only about 64 cases reported to date in the literature (3). Although the presence of several risk factors, such as thorotrast, arsenic, polyvinyl chloride, radiotherapy, and chronic lymphedema, have been reported in angiosarcomas arising at other sites in the body, there is no evidence of a direct relationship between these predisposing factors and primary angiosarcomas of the kidney (2–4). The tumors are usually large, measuring from 3.7 to 30 cm in diameter, and are detected in advanced stages of the disease (3).

Patients frequently present with flank pain and a palpable mass (2–4). As the tumor has a tendency to bleed, patients may also complain of massive hematuria and a retroperitoneal hematoma following spontaneous rupture of the mass (5).

With respect to imaging findings, the tumors have been described as a hypodense mass, with variable peripheral enhancement, or a large necrotic mass (1–5). However, there are very few useful imaging features, suggestive of primary renal angiosarcomas. A previous study described a striated pattern on T2-weighted MRI, as a specific finding suggestive of a primary renal angiosarcoma (7). However, in that case, the mass showed no detectable enhancement (7). In contrast, in this case, we observed early peripheral nodular enhancement accompanied by progressive fill-in on dynamic contrast-enhanced images. These imaging features may also be seen in cases of renal hemangiomas (3, 8). However, as compared to angiosarcomas, renal hemangiomas are relatively small (3). Furthermore, as seen in this case, a tangled mesh of signal-void vascular structures in the periphery of the huge mass on a T2-weighted image could be a useful MRI finding indicative of a primary renal angiosarcoma.

Due to the rarity of this tumor, there are no standard treatment guidelines for primary renal angiosarcomas (1–3, 5). However, most of the reported cases involved patients who underwent radical nephrectomies (2, 3). Radiation therapy and chemotherapy may be subsequently used in localized and metastatic disease, respectively (1, 3). The prognosis is very poor, with more than 70 percent of the reported cases dying within a mean interval of 7.3 months (3).

To summarize, we present a case of primary renal angiosarcoma with extensive hemorrhage and suggest its inclusion in the differential diagnosis of cases of hemorrhagic renal tumors with prominent vasculature.
